# HPLC analysis, mycochemical contents and biological activities of two edible hypogeous ascomycetes: *Tirmania nivea* and *Terfezia boudieri*

**DOI:** 10.1016/j.heliyon.2023.e14331

**Published:** 2023-03-04

**Authors:** Rihab Hammami, Albandary Nasser Alsaloom, Chedia Aouadhi, Ahlam Alrokban, Mohamed Mendili, Ayda Khadhri

**Affiliations:** a University of Tunis El Manar, Faculty of Sciences, Plant, Soil, Environment Interactions Laboratory, Campus Academia, 2092 Tunis, Tunisia; b Biology Department, College of Science, Princess Nourah bint Abdulrahman University, Riyadh 11671, Saudi Arabia; c University of Tunis El Manar, Pasteur Institute of Tunis, Laboratory of Epidemiology and Veterinary Microbiology, Bacteriology and Biotechnology Development Groups, Tunis, Tunisia; d Higher Institute of Biotechnology of Beja, University of Jendouba, Beja, Tunisia

**Keywords:** Biological activity, Truffles, Phenolic compounds, *Terfezia boudieri*, *Tirmania nivea*

## Abstract

Truffle, the hypogeous, ascomycetous macrofungus, has been recognized as a delicacy for centuries, and it is gaining elevated status in the culinary domain. The chemical composition and biological activities of aqueous extract and ground material of two desert Tunisian *Tirmania nivea* and *Terfezia boudieri* were studied for the first time. Using three assays antioxidant activity of the aqueous extract and ground material of the two truffles were investigated. Indeed, the aqueous extract and ground material of *Terfezia boudieri* showed the highest capacity for the DPPH scavenging test (IC_50_ = 0.18 mg/mL) and with regard to the chelating power of iron (IC_50_ = 0.22 mg/mL). At the same time, the highest capacity for iron reduction was recorded in the crude material of *Tirmania nivea*. Besides, the total phenolic, flavonoid, flavanol, tannin, and proanthocyanidin contents of both truffles extracts were determined. The ground material showed the best antibacterial activity for two ascomycetes against seven strains of bacteria. HPLC analysis of aqueous extracts showed that the predominant phenolic compounds in *T. boudieri* and *T. nivea* were gallic acid (33.25%) and myricetin (52.91%). Therefore, these truffles are a source of natural antioxidant and antimicrobial agents and could be used as a potential health food.

## 1 Introduction

Free radicals have gained popularity owing to their central function in different physiological conditions as well as their involvement in a variety of diseases, including many cancers, neurodegenerative disorders, asthma (respiratory diseases), diabetes mellitus, and cardiovascular diseases [1]. Depending on dose and bioavailability, antioxidants have also been shown in several recent studies to have deleterious effects on human health [2]. It is therefore essential to analyze the usefulness of antioxidants in improving human health.

Wild truffles in many countries are commonly used as food and have acquired a high status in the culinary field. In addition, they have been used in human diets and traditional medicine [3]. Furthermore, truffles have a high nutritional value due to their vitamins, mineral salts, protein content, and dietary fiber [4]. Therefore, they are also a source of bioactive compounds such as polyphenols, carotenoids, terpenoids, ergosterol, ascorbic acid, polysaccharides, and phytosterol [5,6]. Truffles secondary metabolites have recently received a lot of attention for their antioxidant, anti-inflammatory, antimicrobial, antimutagenic, and aphrodisiac effects [4,[7], [8], [9], [10]].

As well as truffles, the hypogeous fruiting bodies of Ascomycete have been widely used in traditional world medicine thanks to their nutritional richness and therapeutic effects. Although these ectomycorrhizal truffles that live in symbiosis with the roots of trees are used in the culinary field and have a role in health [7,11]. Lee et al. [5] reported that truffles could be safely used as a potential nutritional source. Today, Ascomycetes hypogeous (truffles) are divided into six Pezizalian families: *Glaziellaceae, Discinaceae-Morchellaceae, Helvellaceae, Tuberaceae, Pezizaceae,* and *Pyronemataceae*, including 38 genera. Instead of the family *Terfeziaceae*, which has been deleted, the best known and most appreciated genera, *Terfezia* and *Tirmania,* have been found to belong to the Pezizaceae, and some members of both genera have shown a monophyletic origin [11]. In addition, numerous research studies have reported numerous biological properties of desert truffles, including antimicrobial, antimalarial, anti-inflammatory, antioxidant, antitubercular, and anticancer activities [4,7,[12], [13], [14]].

Desert truffles, which grow in arid and semi-arid regions such as Tunisia, belong to the genus *Terfezia* and *Tirmania* in general [5]. The analyses included the total content of polyphenol, flavonoid, tannin, flavanol, and proanthocyanidin of aqueous and ground material extracts of *Terfezia boudieri* and *Tirmania nivea*. Therefore, we investigated the antioxidant and antibacterial activities and identified the phenolic compounds using liquid chromatography (HPLC).

## 2 Material and methods

### 2.1 Materials and extract preparation

The hypogenous ascomycetes: *Tirmania nivea* (Desf.) Trappe and *Terfezia boudieri* Chatin were collected from the Tataouine and Medenine regions of Tunisia (32°53’57” N, 10°26’15” E and 33°20’17” N, 10°29’53” E) in March 2018. The identification of specimens is based essentially on the determination keys [[15], [16], [17], [18]]. The confirmation of this identification was made by the French mycologist Mr. Pierre ROUX.

*T. nivea* and *T. boudieri* were freeze-dried for two days, after which they were grinded. Both truffles are extracted using boiling water. Ultrasonic extraction was conducted for over 1 h. The water extracts were freeze-dried and the extracts were preserved at 4 °C until further analysis was performed.

### 2.2 Phenolic profile

#### 2.2.1 Determination of the total phenolic content

Total phenolic content was determined using the Follin-Ciocalteu method [19]. 500 μL of distilled water was mixed with 125 μL of diluted extracts (1 mg/mL) of two desert truffles (*T. nivea* and *T. boudieri*) with 125 μL of Follin-Ciocalteu reagent. Following agitation of the mixture, it was allowed to stand for 5 min. Then 1 mL of distilled water and 1250 μL of Na_2_CO_3_ (7%) were added. The absorbance at 760 nm was measured after a 90 min incubation period in the dark. Gallic acid solutions (0–400 mg/mL) were used for the standard calibration curve. The results are expressed in gallic acid equivalents (GAE) per gram of dry weight.

#### 2.2.2 Determination of the total flavonoid content

The method of Djeridane et al. [20] was used to determine the total flavonoid content. 1 mg of powdered truffle material or 1 mg of fungal extract (aqueous) was dissolved in 1 mL of distilled water. Then, 1 mL of each diluted extract was mixed with 150 μL of freshly prepared aluminum chloride solution AlCl_3_ (10%). After 5 min of resting at room temperature, 500 μL of sodium hydroxide (1 M) is applied to the mixture, bringing the total volume to 2500 μL with distilled water. This solution's absorbance is measured at 510 nm. The results are expressed as mg of catechin equivalent per gram of dry weight (mg CE/g DW).

#### 2.2.3 Determination of the total flavanol content

The flavanol content was measured following Huang et al. [21] method, which relies on the formation of a complex between flavanol and aluminum chloride. 2 mL of extract were added with 2 mL of AlCl_3_ ethanolic solution (2%). 3 mL of NaNO_2_ were added. After 2 h and 30 min incubation at 20 °C. After minutes, the absorbance was measured at 440 nm. Quercetin (1.8 mg/mL) equivalent per gram of dry weight is used to express the results (mg QE/g DW).

#### 2.2.4 Determination of the total proanthocyanidin content

The butanol-HCl assay was used to evaluate the concentration of proanthocyanidin [22]. An aliquot of 0.5 mL of *Tirmania nivea* and *Terfezia boudieri* extracts were combined with 3 mL of butanol-HCl (95:5; v/v) and 0.1 mL of iron sulfate (2 g/mL) in a mixture. The whole was allowed to stand at 90 °C for 1 h before the absorbance was measured at 530 nm. The catechin equivalent per gram of dry weight (mg CE/g DW) was used to calculate the proanthocyanidin content.

#### 2.2.5 Determination of the total tannin content

Using the method of Sun et al. [23] total tannin content was determined. 3 mL of vanillin methanol solution (4%) and 1.5 mL of HCl solution (70%) were added to 50 mL of different diluted extracts. After a 15-min period of rest, the absorbance was measured at 500 nm. Then, results were expressed as milligrams of tannic acid equivalent per gram of dry weight (mg TAE/g DW). Tannic acid standard curve was established between 0 and 400 mg/mL.

### 2.3 Antioxidant capacity assays

#### 2.3.1 DPPH radical scavenging assay

According to the method of Hatano et al. [24], the DPPH· radical scavenging assay was carried out. 1 mL of *T. nivea* and *T. boudieri* extracts was added to 0.5 mL of DPPH· ethanolic solution (0.2 mM). The absorbance was measured at 517 nm after the whole mixture had been allowed to rest for 30 min at room temperature. The inhibition percent (IP %) of the free radical DPPH was determined as follows:

IP (%) = ((A_blank_– A_sample_)/A_blank_) × 100, where A_blank_ is the absorbance of the control reaction and A_sample_ is the absorbance in the presence of *T. nivea* and *T. boudieri* extracts.

#### 2.3.2 Iron chelating power

With slight modifications, the ferrous ion-chelating power was measured using the method of Decker & Welch [25]. 0.1 mL of *T. nivea* and *T. boudieri* extracts at different concentrations (from 0 to 400 mg/mL) was added to 0.05 mL of FeCl_2_, 4H_2_O (2 mM). After shaking for 5 min, 1 mL of ferrozine (5 mM) and 2.75 mL of distilled water were poured into the mixture. Then, the solution was shaken again and allowed to rest at room temperature for 10 min. Later, the absorbance of the samples was read at 562 nm and the results were compared to those of negative control (extract which was replaced by methanol) and positive control (EDTA).

#### 2.3.3 Reducing power assay

The reducing power was estimated using the method of Oyaizu [26]. 200 μL extracts of *T. nivea* and *T. boudieri* were combined with 500 μL sodium phosphate buffer (200 mM, pH = 6.6) and 2.5 mL potassium ferricyanide (1%). At 50 °C, the mixture was allowed to settle for 20 min. The mixture was then centrifuged at 650×*g* for 10 min with 2.5 mL of trichloroacetic acid (10%) added. After that, 500 μL of supernatant were combined with 500 μL of deionized water and 100 μL of ferric chloride (0.1%). At 700 nm, the absorbance was taken and the following formula was used to calculate the percent of iron-chelating ability:

= (A _control_ – A _extract_ / A _control_) × 100, where, A _control_ = Absorbance of control reaction; A _extract_ = Absorbance of *T. nivea* and *T. boudieri* extract

### 2.4 Antioxidant assessment of solid substances by QUENCHER method

The QUENCHER method was used to apply total phenolic compounds, flavonoid, tannin, and proanthocyanidin content as well as DPPH, iron-chelating power, and iron-reducing power assays directly to the solid ground of *T. nivea* and *T. boudieri* [27]. To begin, the samples were diluted with an innocuous substance, microcrystalline cellulose. Using 10 mg of ground *T. nivea* and *T. boudieri*, all analyses were carried out in the same way as stated for extracts. In all assays, the produced mixtures were vortexed for 15 s before being shaken at 250 rpm in the dark for 2 h and then centrifuged (1960×*g* for 5 min). For total phenolic compounds, flavonoid, proanthocyanidin, and tannin contents, optical absorbance of clear supernatants was measured at 760 nm, 430 nm, 530 nm, and 500 nm, respectively, and was measured at 517 nm for DPPH, 560 nm for iron chelating power, and 700 nm for iron-reducing power for the antioxidant capacity assays.

### 2.5 Antibacterial activity

Using the disk diffusion assay, antimicrobial activity was first determined by measuring the diameter of the growth-inhibition zone in millimeters for the test organisms compared to the controls [28]. In addition, using a broth dilution method established by Cosentino et al. [29], the minimum inhibitory concentration (MIC) and minimum bactericidal concentration (MBC) of each tested extract were obtained. The MIC was the lowest concentration that caused a significant reduction in inoculum viability (>90%), whereas the MBC was the concentration that destroyed 99.9% or more of the starting inoculum.

### 2.6 Determination of phenolic compounds by HPLC method

An Agilent 1100 series HPLC system with an online degasser (G 1322A), a quaternary pump (G 1311A), a thermostatic autosampler (G 1313A), a column heater (G 1316A), and a diode array detector was used to separate phenolic compounds (G 1315A). Instrument control and data analysis were carried out using Agilent HPLC Chemstation 10.1 edition through Windows 2000. The separation was carried out on a reverse-phase ODS C18 column (4 mm, 250 × 4.6 mm, Hypersil) used as a stationary phase at ambient temperature. The mobile phase was comprised of acetonitrile (solvent A) and water with acetic acid as solvent B (0.2 mL/100 mL water). The flow rate was kept at 0.5 mL/min. The gradient program was as follows: 15% A/85% B 0–12 min, 40% A/60% B 12–14 min, 60% A/40% B 14–18 min, 80% A/20% B 18–20 min, 90% A/10% B 20–24 min, and 100% A 24–28 min. The injection volume was 20 μl and peaks were monitored at 280 nm. Peak identification was obtained by comparing the retention time and the UV spectra of the fraction phenolic chromatogram with those of pure standards, which were purchased from Sigma (St. Louis, MO, USA).

### 2.7 Statistical analysis

All the analyses were repeated three times, and the results were based on the average of three replicates. Values were expressed as mean ± standard deviations. MS Excel 2010 and SPSS software programs were used to perform statistical analysis. Correlation coefficients (r) between the total phenolic compounds, flavonoid, tannin, and proanthocyanidin contents and the DPPH radical scavenging capacity, reducing power, and iron-chelating power were calculated. Using IBM SPSS 20, Pearson's correlation coefficients were examined, and intriguing correlations (P < 0.01) were discovered.

## 3 Results and discussion

### 3.1 Total phenolic compounds, flavonoid, flavanol, tannin and proanthocyanidin contents

Oxidative stress is linked to several diseases, including diabetes and cancer, and can disrupt human function [30]. Many researchers had investigated at natural products as a source of bioactive compounds, such as those found in edible truffles. There are also potential natural product resources [30]. In the current research, the total phenolic compounds, flavonoid, flavanol, tannin, and proanthocyanidin contents of *T. boudieri* and *T. nivea* extracts were determined. The number of secondary metabolites in truffles extracts varied according to the type of extract and between species, as shown in Table 1. In fact, *T. boudieri* extracts were richer in secondary metabolites than the white truffle *T. nivea* ones. Total polyphenol content was found to range from 12.5 mg GAE/g DW in water extract to 36.43 mg GAE/g DW in QUENCHER extract. The highest flavonoid and tannin contents were recorded in QUENCHER extracts of *T. boudieri* (13.2 mg CE/g DW and 8.12 mg TAE/g DW, respectively).

**Table 1:** Phenolic concentrations of the water and QUENCHER extracts of *T. boudieri* and *T. nivea*.

Truffle species	Extract	Total polyphenol (mg GAE/g DW)	Total flavonoid (mg CE/g DW)	Flavanol (mg QE/g DW)	Total tannin (mg TAE/g DW)	Proanthocyanidin (mg CE/g DW)
***T. boudieri***	**Water**	17.15 ± 0.08^c^	5.63 ± 0.07^c^	0.32 ± 0.01^a^	4.34 ± 0.05^b^	1.78 ± 0.01^a^
	**QUENCHER**	36.43 ± 0.09^a^	13.2 ± 0.05^a^	0.18 ± 0.01^b^	8.12 ± 0.07^a^	1.73 ± 0.01^a^
***T. nivea***	**Water**	12.5 ± 0.03^d^	6.74 ± 0.03^b^	0.35 ± 0.03^a^	1.23 ± 0.02^c^	1.37 ± 0.02^b^
	**QUENCHER**	24.90 ± 0.06^b^	2.5 ± 0.02^d^	0.13 ± 0.00^b^	2.26 ± 0.01^c^	1.43 ± 0.03^b^

Data are presented as the mean ± standard deviation. CE, (+)-catechin equivalent; DW, dry weight; GAE, gallic acid equivalent; QE, quercetin equivalent; TAE, tannic acid equivalent.

The results showed that both truffles contain phenolic compounds. Furthermore, these results agree with previous work that has shown the richness of truffles in secondary metabolites [3,[31], [32], [33]]. Other studies have found that *T. nivea* water extracts contain a high amount of polyphenol and flavonoid [7]. Although *T. boudieri* had the highest amount of polyphenol (17.15 mg GAE/g DW) and flavonoid (5.63 mg QE/g DW) compared to other truffles extracts such as *Auricularia auricula-judae* (Bull.) Quèl (2.90 mg GAE/g DW and 1.62 mg QE/g DW) [3].

Indeed, our results are in accordance with those previously reported by Gouzi et al. [13]. They indicated that wild edible desert truffles are a valuable source of various secondary metabolites such as phenolic compounds, carotenoids, and anthocyanin contents. Hence, the Tunisian truffles show a high content of phenolic compounds [7].

The ground material has the highest phenolic content as well as the important phenolic compounds and flavonoid content found in *T. boudieri.* The QUENCHER method is a novel technique that must be used without the use of any solvents. It is based on solid-solid dilution using cellulose. Cellulose powder is frequently used to dilute plant material since cellulose is devoid of free radicals [29]. In addition, the QUENCHER method has higher levels of phenolic compounds than the aqueous extract. Furthermore, previous research has shown that the QUENCHER process was higher values than polar and apolar extracts [6,34].

Indeed, these compounds may have important antioxidant activity. Numerous studies have determined the polyphenol and flavonoid content and antioxidant activities of macrofungus [5,6,10,14,35]. According to Islam, Yu & Xu [30] phenolic compounds can protect the human body from free radicals. In addition, they have been linked to an increase in the prevention of various human disorders, including Alzheimer's disease [30]. Phenolic compounds and flavonoids have antioxidant properties and other biological activities [19,20,36].

Several studies have reported that the antioxidant activity of hypogeous macrofungus is due to their richness in secondary metabolites, including phenolic compounds while scavenging free radicals and/or chelating metal ions [7,37]. Besides, several studies have shown that truffles have a wide range of biological activity, including antibacterial activity. In this work, this activity may be the origin of proanthocyanidin, which was determined as an active antibacterial agent against different pathogens [38,39].

The antioxidant potential of *Tirmania nivea* correlates with the combination of anthocyanin and carotenoid content as well as ascorbic acid content [5]. Also, they approved that catechin was responsible for the high scavenging effect of *Terfezia boudieri* [5]*.*

### 3.2 Antioxidant activity

The antioxidant activities of *T. boudieri* and *T. nivea* in both extracts are shown in Table 2. Indeed, the antioxidant properties of both truffles were assessed as DPPH. radical scavenging, iron-reducing power, and iron-chelating power. In fact, the highest DPPH˙ scavenging capacity was observed in the water extract of *T. boudieri* (IC_50_ = 0.18 mg/mL), followed by the water extract of *T. nivea* (IC_50_ = 0.25 mg/mL). Moreover, an important iron-chelating power was observed in the QUENCHER extract of *T. boudieri* (IC_50_ = 0.22 mg/mL). The highest reducing power for *T. nivea* was shown in the QUENCHER extract. (IC_50_ = 1.67 mg/mL). As a result of *T. boudieri* and *T. nivea,* we can conclude that the water extract and QUENCHER approach studied have significant antioxidant activity. In addition, the same study showed that truffles have high antioxidant potential against DPPH, ferric reducing, and superoxide anion [13].

**Table 2:** Radical Scavenging Activity, Reducing Power and Metal-Chelating Power of the water and QUENCHER extracts of *T. boudieri* and *T. nivea*.

Antioxidant activity (IC50, mg/mL)				
Truffle species	Extract	DPPH	Reducing Power	Iron Chelation
***T. boudieri***	**Water**	0.18 ± 0.01	2.44 ± 0.03	0.51 ± 0.01
	**QUENCHER**	0.36 ± 0.02	2.20 ± 0.04	0.22 ± 0.01
***T. nivea***	**Water**	0.25 ± 0.01	3.85 ± 0.06	0.38 ± 0.02
	**QUENCHER**	0.55 ± 0.01	1.67 ± 0.08	0.87 ± 0.03
**BHT**		0.017 ± 0.03		
**EDTA**				0.05 ± 0.04
**Vit C**			0.035 ± 0.02	

IC_50_, half-maximal inhibitory concentration.

Also, our results agree with those of Beara et al. [4] who proved that both ascomycetes, *Tuber aestivum* and *Tuber magnatum* have antioxidant activity. Furthermore, methanolic extracts of these two ascomycetes showed cytotoxicity against some tumor cells and preponderant activity of aqueous extracts against breast adenocarcinoma. The QUENCHER method carefully evaluated the increase in antioxidant activity, but the solvent extraction method failed to achieve this [27]. This is probably due to the low solubility.

### 3.3 Antibacterial activity

The screening of water antibacterial activity and QUENCHER extracts of two truffles were evaluated based on IZs, MICs, and MBCs diameters against common Gram-positive and Gram-negative bacteria (Table 3). According to the results, the truffle extracts had varying degrees of antibacterial activity against all the strains tested. In fact, QUENCHER extracts (MIC: 0.025 g/mL) of two truffles showed interesting antimicrobial activities against all tested strains as compared to water extracts (MIC: 0.05 g/mL).

**Table 3:** Diameter of Inhibtion Zones, Minimum Inhibitory Concentrations and Minimum Bactericidal Concentrations of the water and QUENCHER extracts of *T. boudieri* and *T. nivea*.

		Diameter of Inhibtion Zones (mm)				MIC (g/mL)				MBC (g/mL)			
		Water		QUENCHER		Water		QUENCHER		Water		QUENCHER	
Tested Strains	Gentamicin	*T. boudieri*	*T. nivea*	T. boudieri	*T. nivea*	*T. boudieri*	*T. nivea*	*T. boudieri*	*T. nivea*	*T. boudieri*	*T. nivea*	*T. boudieri*	*T. nivea*
***E. coli* ATCC 8739**	24	12	11	13	13	0.05	0.05	0.025	0.025	0.1	0.1	0.05	0.05
***S. typhimurium* NCTC 6017**	23	11	12	14	14	0.05	0.05	0.025	0.025	0.1	0.1	0.05	0.05
***A. hydrophila* EI**	23	12	11	13	12	0.05	0.05	0.025	0.025	0.1	0.1	0.05	0.05
***P. aeruginosa* ATCC 27853**	21	12	13	14	13	0.05	0.05	0.025	0.025	0.1	0.1	0.05	0.05
***S. aureus* ATCC 29213**	20	11	12	12	12	0.05	0.05	0.025	0.025	0.1	0.1	0.05	0.05
***L. monocytogenes* ATCC 7644**	18	12	13	13	14	0.05	0.05	0.025	0.025	0.1	0.1	0.05	0.05
***B. cereus* ATCC1247**	21	11	11	13	13	0.05	0.05	0.025	0.025	0.1	0.1	0.05	0.05

ATCC, American Type Culture Collection; MBC, minimum bactericidal concentration; MIC, minimum inhibitory concentration; NCTC, National Collection of Type Cultures.

Generally, antimicrobial activity is the third generalized therapeutic effect reported for macrofungus, including antibacterial, antifungal, antiparasitic, and antiviral activity. Indeed, more than 200 species of macrofungus are thought to have antimicrobial properties [40].

Nevertheless, it has been previously mentioned that desert truffles have antibacterial activity against Gram-positive and Gram-negative pathogenic bacteria like *Pseudomonas aeruginosa* and *Staphylococcus aureus* [41]. Furthermore, Gouzi et al. [42] used agar well diffusion and kinetic bacterial growth curves techniques to examine the excellent activity of the aqueous extracts against various pathogens. Besides, they have found that Desert Truffles (*T. Tirmania*) showed strong activity against *Pseudomonas aeruginosa* and *Staphylococcus aureus* [13,41]*.*

Hamza et al. [7] also reported that the aqueous extract of *T. nivea* has antimicrobial activity against a wide range of Gram-positive and negative bacteria, as well as that extracts from this desert truffle could be used to control the microbiological quality of processed foods.

### 3.4 HPLC analysis

HPLC was used to characterize the chemical composition of extracts. Thirty phenolic compounds were identified in both truffles. While 28 of them were identified in *T. boudieri*, 6 of them were identified in *T. nivea* (Table 4). Gallic acid was the most abundant phenolic compounds in *T. boudieri* with a percentage of 33.25%, followed by myricetin (6.75%) and quinic acid derivative (5.22%). The most abundant phenolic compounds in *T. nivea* were identified as myricetin (52.91%) and isorhamnetin (40.23%).

**Table 4:** HPLC identification of phenolic compounds of hypogenous Ascomycota truffles.

RT	Compounds	*T. boudieri*	*T. nivea*
4.93	Aconitic acid derivative	0.17	nd
4.80	p-Coumaroylmalic acid	2.31	0.39
5.25	Caffeic hexosemalonate-carboxyl	1.03	nd
5.30	Dimer of ferulic acid deoxyhexoside	2.76	nd
5.49	Caffeic acid hexoside	**3.78**	nd
5.69	**Gallic acid**	**33.25**	nd
6.13	Malic acid	**7.71**	0.28
6.31	Malic acid derivative	1.99	nd
6.56	p-Coumaric acid	0.63	nd
6.91	Caffeic acid	**3.09**	nd
7.41	Citric acid	**3.90**	nd
7.58	Quinic acid	0.79	nd
7.79	Catechin hexoside	0.39	nd
7.85	Quinic acid derivative	**5.22**	nd
9.00	Dimer of cinnamic acid	0.33	nd
9.07	Protocatechuic acid hexoside	0.20	nd
9.93	Dimer of protocatechuic acid	0.53	nd
10.57	Protocatechuic acid derivative	0.54	nd
11.34	Cinnamic acid derivative	0.40	nd
11.59	Dimer of cinnamic acid	0.12	nd
12.04	p-Hydroxybenzoic	0.15	nd
13.08	Protocatechuic acid	0.40	nd
13.72	Vanillic acid desoxyhexoside	0.20	nd
17.80	Synergic acid	0.18	nd
18.45	Shikimic-desoxyhexosyl-hexoside	0.19	nd
20.52	Catequin hexosemalonate	nd	0.97
26.66	Dimer of caffeic acid hexoside	nd	2.45
26.95	Cirsimaritin	0.56	nd
30.39	Myricetin	**6.75**	52.91
30.76	Isorhamnetin	2.66	40.23

nd: Not detected.

Generally, the phenolic compounds detected correspond to a typical phenolic profile of edible truffles [4,14]. Similar results have been reported by Yahia et al. [14] on wild macrofungus in which different organic acids such as gallic, shikimic, malic, coumarilic, fumaric acids have been identified. Overall, these results suggest that the major compounds, gallic acid, myricitin, and isoramnethin, detected in the phenolic profile of these two ascomycetes studied are at the origin of the observed biological activities such as antioxidant and antibacterial activity [[43], [44], [45], [46]].

The bioactive compounds detected in edible truffles in this study, along with low levels of fat, protein, and carbohydrates, contribute to the nutritional value and health of these species. However, to the best of our knowledge, this is probably the first chemical investigation of the phenolic profile of these two Tunisian truffles, *Terfezia boudieri,* and *Tirmania nivea*, that might provide a valuable basis for the eventual approval of their use as a health food.

## 4 Conclusion

In conclusion, this study showed that the tested truffles have high quantities of phenolic compounds and possess antioxidant and antimicrobial activities. They can be of great importance in the treatment of various diseases, and they can be used in nutrition to help the organism to protect against oxidation.

## Authors’ contribution statement

Rihab Hammami: Conceived and designed the experiments; Performed the experiments; Analyzed and interpreted the data; Wrote the paper.

Albandary Nasser Alsaloom, Chedia Aouadhi, Ahlam Alrokban: Performed the experiments; Wrote the paper.

Mohamed Mendili: Conceived and designed the experiments; Performed the experiments.

Ayda Khadhri: Conceived and designed the experiments; Performed the experiments; Analyzed and interpreted the data; Contributed reagents, materials, analysis tools or data; Wrote the paper.

## Funding statement

Ayda Khadhri was supported by Tunisian Ministry of Higher Education and Scientific Research.

Albandary Nasser Alsaloom was supported by 10.13039/501100022230Princess Nourah bint Abdulrahman University.

## Data availability statement

Data included in article/supp. material/referenced in article

## Declaration of interest statement

The authors declare no conflict of interest.

## Abbreviations

CE: (+)-catechin equivalent
DPPH: 2,2-diphenyl-1-picrylhydrazyl
DW: dry weight
GAE: gallic acid equivalent
HPLC: high performance liquid chromatography
IC50: half-maximal inhibitory concentration
IZ: inhibition zone
MBC: minimum bactericidal concentration
MIC: minimum inhibitory concentration
PI: percentage inhibition
QE: quercetin equivalent
QUENCHER: (QU: quick, E: easy, N: new, CHE: cheap, R: reproducible)
TAE: tannic acid equivalent
